# Supplementary material for: Activation of the cell wall integrity pathway negatively regulates TORC2-Ypk1/2 signaling through blocking eisosome disassembly in Saccharomyces cerevisiae
Source: Commun Biol. 2024 Jun 11;7:722. doi: 10.1038/s42003-024-06411-2 (PMC11166964; doi:10.1038/s42003-024-06411-2)
Supplement: Supplementary file 2 — Supplementary Information [file 42003_2024_6411_MOESM2_ESM.pdf]

**Supplementary Table 1.** ***Saccharomyces cerevisiae*** **strains used in this study**

| **Strain** | **Relevant genotype/description** | **Source/Reference** |
|---|---|---|
| BY4741 | *MAT***a** *his3Δ1 leu2Δ0 met15Δ0 ura3Δ0* | Lab stock |
| *fps1Δ* | BY4741, *fps1Δ::KanMX4* | Invitrogen |
| *erg2Δ* | BY4741, *erg2Δ::KanMX4* | Invitrogen |
| *erg3Δ* | BY4741, *erg3Δ::KanMX4* | Invitrogen |
| *erg6Δ* | BY4741, *erg6Δ::KanMX4* | Invitrogen |
| *vps29Δ* | BY4741, *vps29Δ::KanMX4* | Invitrogen |
| *vps35Δ* | BY4741, *vps35Δ::KanMX4* | Invitrogen |
| *ORM1-TAP* | BY4741, *ORM1-TAP::HIS3MX6* | Open Biosystems |
| DL376 | *MAT***a** *leu2-3, 112 trp1-1 ura3-52 his4 can1 pkc1Δ::LEU2* | 79 |
| *wsc1Δ* | BY4741, *wsc1Δ::KanMX4* | Invitrogen |
| *mid2Δ* | BY4741, *mid2Δ::KanMX4* | Invitrogen |
| *wsc1Δmid2Δ* | BY4741, *wsc1Δ::KanMX4 mid2Δ::*$his5^+$ | This study |
| *PKC1* | BY4741, *PKC1::LEU2* | This study |
| $PKC1^{L54S\_4C/S}$ | BY4741, $PKC1^{L54S\_4C/S}$*::LEU2* | This study |
| *bck1Δ* | BY4741, *bck1Δ::KanMX4* | Invitrogen |
| *mpk1Δ* | BY4741, *mpk1Δ::KanMX4* | Invitrogen |
| *SUR7-mCherry* | BY4741, *SUR7-mCherry::URA3* | This study |
| *SUR7-mCherry SLM1-GFP* | BY4741, *SUR7-mCherry::URA3 SLM1-GFP::KanMX* | This study |
| *SLM1-GFP* | BY4741, *SLM1-GFP::KanMX* | This study |
| *LSP1-mCherry* | BY4741, *LSP1-mCherry::NatNT2* | This study |
| *AVO3-3GFP* | BY4741, *AVO3-3GFP::LEU2* | This study |
| *pil1Δ* | BY4741, *pil1Δ::KanMX4* | Invitrogen |
| *pil1Δlsp1Δ* | BY4741, *pil1Δ::KanMX4 lspΔ::CgLEU2* | This study |
| *gas1Δ* | BY4741, *gas1Δ::KanMX4* | Invitrogen |

## Supplementary Table 2. List of plasmids used in this study

| Plasmid | Description | Source/Reference |
| --- | --- | --- |
| pFR22 | YEp352 (2μ type, *URA3* marker) harboring *PKC1* | 80 |
| pFR74 | YEp352 (2μ type, *URA3* marker) harboring *PKC1*$^{S1143A}$ | 80 |
| YCpLG | CEN type, *LEU2* marker, *GAL1* promoter | 81 |
| YCpLG-*WSC1* | YCpLG harboring *WSC1* | This study |
| YCpLG-*MID2* | YCpLG harboring *MID2* | This study |
| YCpLG-*PKC1* | YCpLG harboring *PKC1* | 82 |
| YCpLG-*PKC1*$^{R398P}$ | YCpLG harboring *PKC1*$^{R398P}$ | 82 |
| pRS416-*pGAL1-HA-RHO1* | pRS416 (CEN type, *URA3* marker) harboring *pGAL1-HA-RHO1* | This study |
| pRS416-*pGAL1-HA-RHO1*$^{Q68L}$ | pRS416 (CEN type, *URA3* marker) harboring *pGAL1-HA-RHO1*$^{Q68L}$ | This study |
| pRS306-*SUR7-mCherry* | pRS306 (integrate-type, *URA3* marker) harboring *SUR7-mCherry* | This study |
| pRS30Gen-*SLM1-GFP* | pRS30Gen (integrate-type, *KanMX* marker) harboring *SLM1-GFP* | This study |
| pRS30Nat-*LSP1-mCherry* | pRS30Nat (integrate-type, *NatNT2* marker) harboring *LSP1-mCherry* | This study |
| pRS305-*PKC1*$^{\Delta 751\text{-}1151}$ | pRS305 (integrate-type, *LEU2* marker) harboring *PKC1*$^{\Delta 751\text{-}1151}$ | This study |
| pRS305-*PKC1*$^{\Delta 751\text{-}1151}$*(L54S_4C/S)* | pRS305 (integrate-type, *LEU2* marker) harboring *PKC1*$^{\Delta 751\text{-}1151}$*(L54S_4C/S)* | This study |
| pRS305-*AVO3-3GFP* | pRS305 (integrate-type, *LEU2* marker) harboring *AVO3-3GFP* | 36 |
| pRS413-*pGAL1-MID2* | pRS413 (CEN type, *HIS3* marker) harboring *pGAL1-MID2* | This study |
| pRS416-*PIL1-13myc* | pRS416 (CEN type, *URA3* marker) harboring *PIL1-13myc* | This study |
| pRS416-*PIL1*$^{S230A/T233A}$*-13myc* | pRS416 (CEN type, *URA3* marker) harboring *PIL1*$^{S230A/T233A}$*-13myc* | This study |
| pRS423-*YPK1-3HA* | pRS423 (2μ type, *HIS3* marker) harboring *YPK1-3HA* | This study |
| pRS423-*YPK1*$^{D242A}$*-3HA* | pRS423 (2μ type, *HIS3* marker) harboring *YPK1*$^{D242A}$*-3HA* | This study |

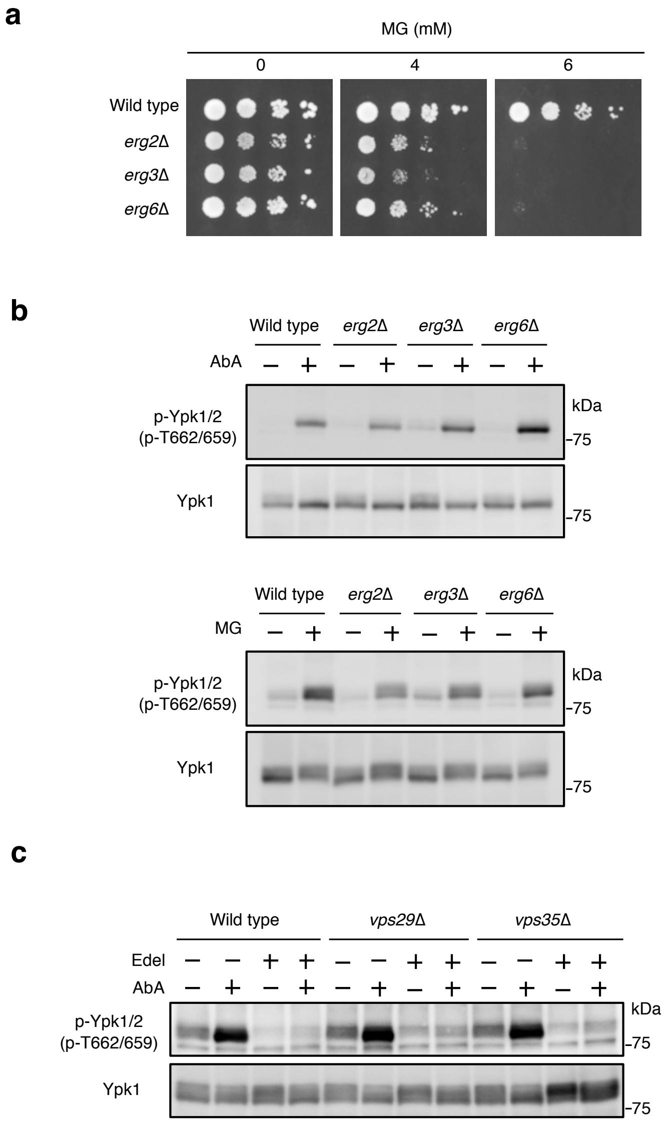


**Supplementary Figure 1.** (A) Wild-type (BY4741), *erg2*Δ, *erg3*Δ, and *erg6*Δ cells were cultured in SD medium at 28°C until the log phase of growth and serially diluted (1:10) using 0.85% NaCl solution. 5 µl each cell suspension was spotted onto SD agar plates with MG and incubated at 28°C for three days. (B) Wild-type (BY4741), *erg2*Δ, *erg3*Δ, and *erg6*Δ cells were cultured in SD medium until the log phase of growth and treated with 1.25 µM AbA or 15 mM MG for 60 min. Phosphorylated Ypk1/2 or Ypk1 were detected with anti-phosphorylated $Ypk1^{T662}$ and $Ypk2^{T659}$ or anti-Ypk1. (C) Wild-type (BY4741), *vps29*Δ, and *vps35*Δ cells were cultured in SD medium until the log phase of growth and pretreated with 5 µM edelfosine for 15 min, and were then further grown for another 60 min after addition of 1.25 µM AbA. The phosphorylated Ypk1/2 or Ypk1 was detected as described in (B).

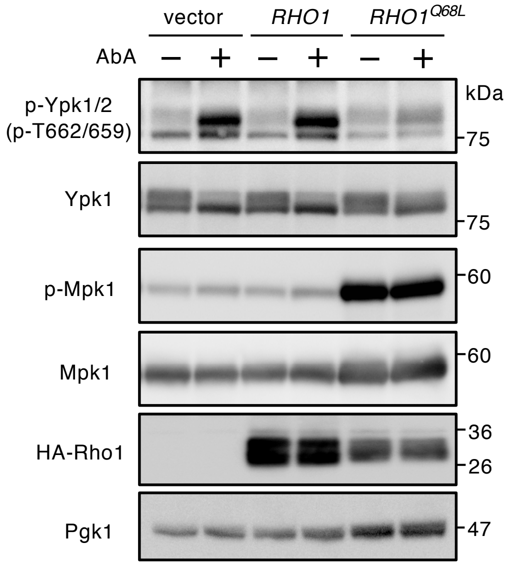


**Supplementary Figure 2.** Wild-type (BY4741) cells carrying an empty vector, pRS416-*pGAL1-HA-RHO1*, or pRS416-*pGAL1-HA-RHO1*$^{Q68L}$ were cultured in raffinose medium until the log phase of growth, and galactose was added to the medium to a concentration of 2%. After incubation for 2.5 h, cells were treated with 1.25 μM AbA for 60 min. Phosphorylated Ypk1/2, Ypk1, phosphorylated Mpk1, Mpk1, HA-Rho1, or Pgk1 was detected with their respective antibodies.

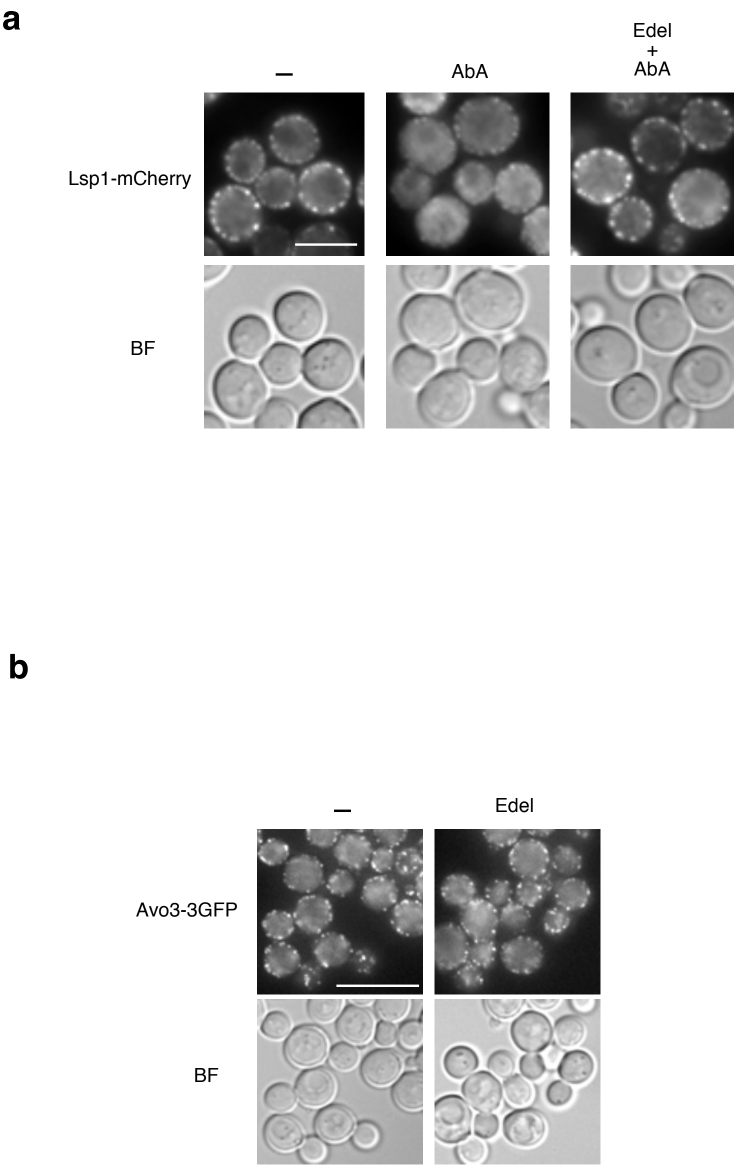


**Supplementary Figure 3.** (A) *LSP1-mCherry* cells were cultured in SD medium until the log phase of growth and pretreated with 5 μM edelfosine for 15 min, and further grown for another 60 min after addition of 1.25 μM AbA. Lsp1-mCherry was observed using a fluorescence microscope. Scale bar: 5 μm. (B) *AVO3-3GFP* cells were cultured in SD medium until the log phase of growth and treated with 5 μM edelfosine for 60 min. Avo3-3GFP was observed using a fluorescence microscope. Scale bar: 10 μm.

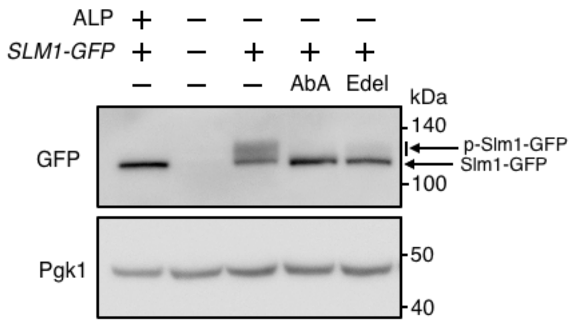


**Supplementary Figure 4.** *SLM1-GFP* cells were cultured in SD medium until the log phase of growth and treated with 1.25 µM AbA or 5 µM edelfosine for 60 min. A portion of the untreated control sample was treated with calf intestinal alkaline phosphatase. Slm1-GFP was detected using GFP antibody.

**Supplementary Figure 5.** Uncropped western blot images for all figures.

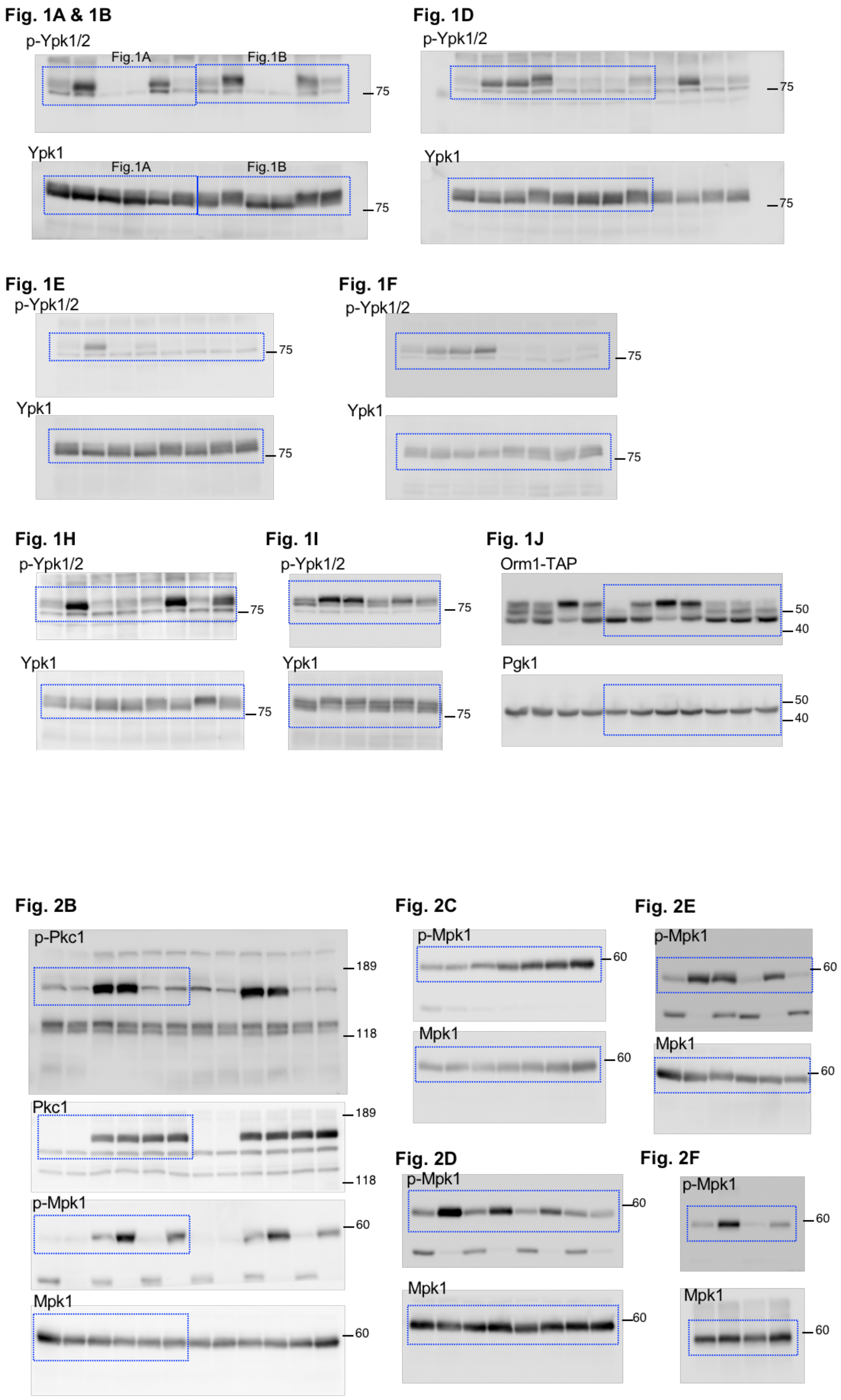

Fig. 3A

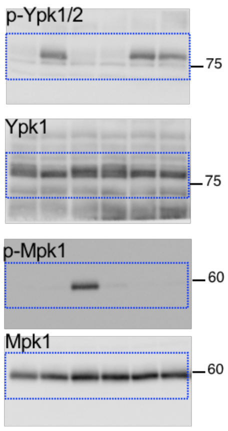


Fig. 3B

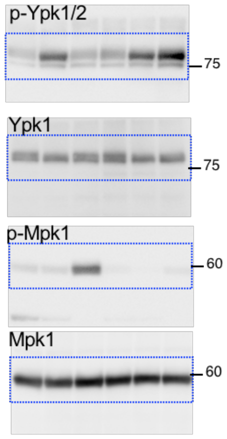


Fig. 3C

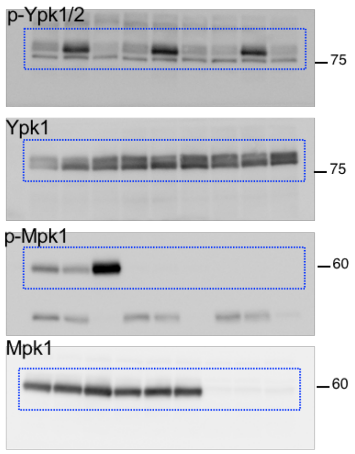


Fig. 3D

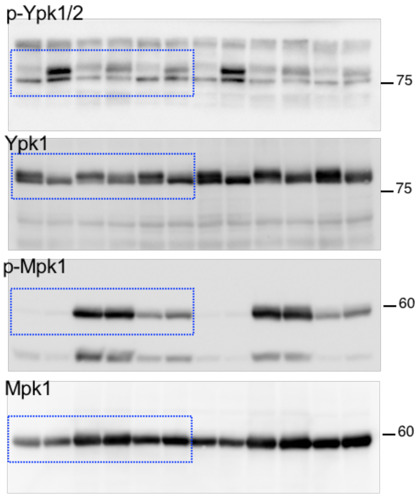


Fig. 3E

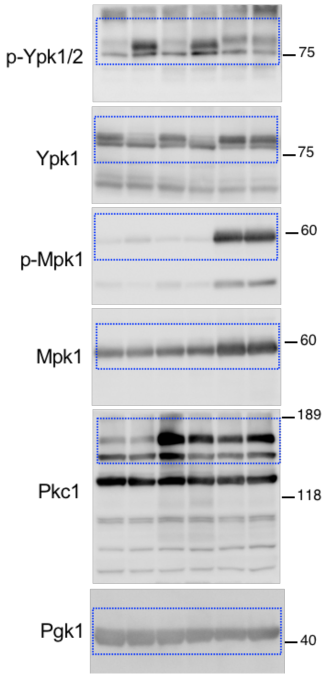

Fig. 5A

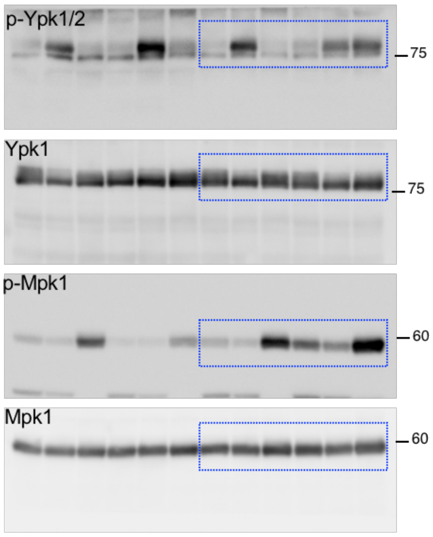


Fig. 5B

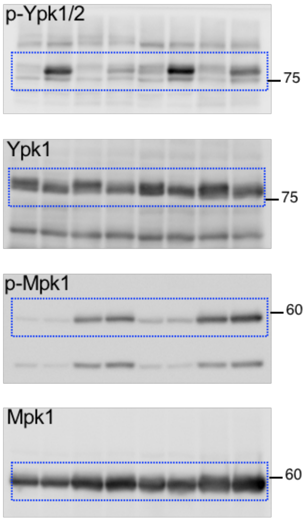


Fig. 5D

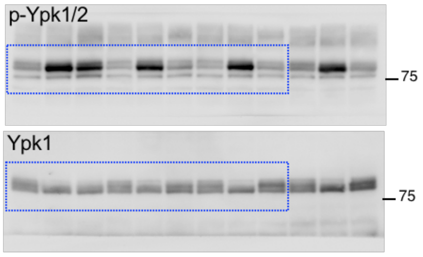

**Fig. 6A**

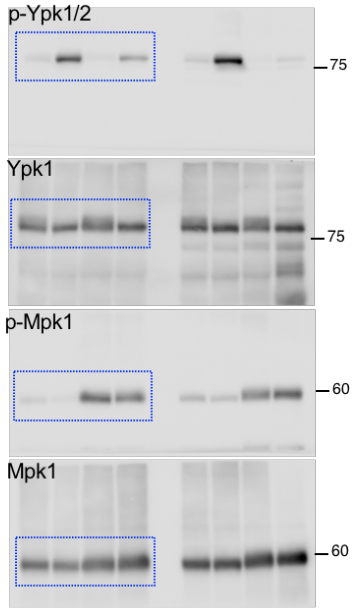


**Fig. 6E**

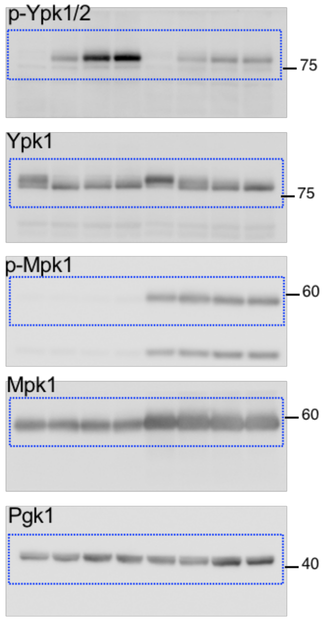


**Fig. 6B**

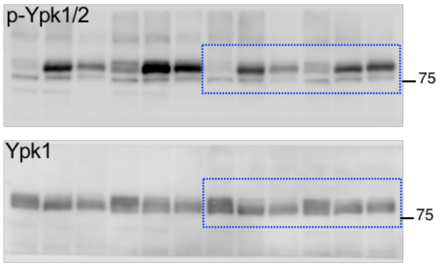


**Fig. S1B**

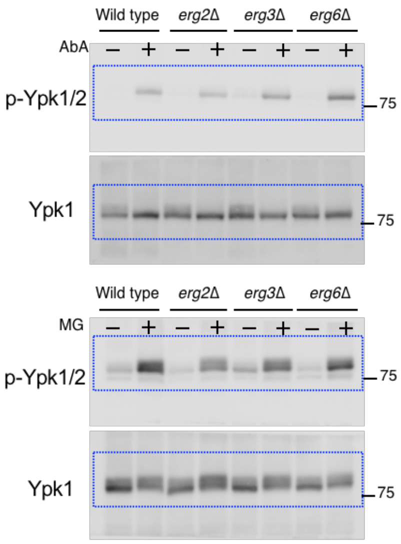


**Fig. S1C**

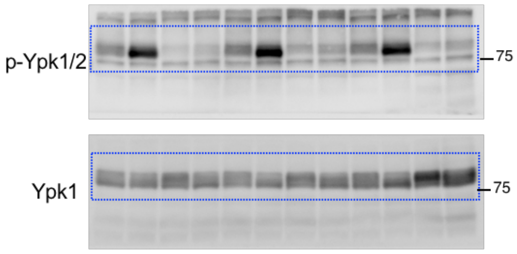

**Fig. S2**

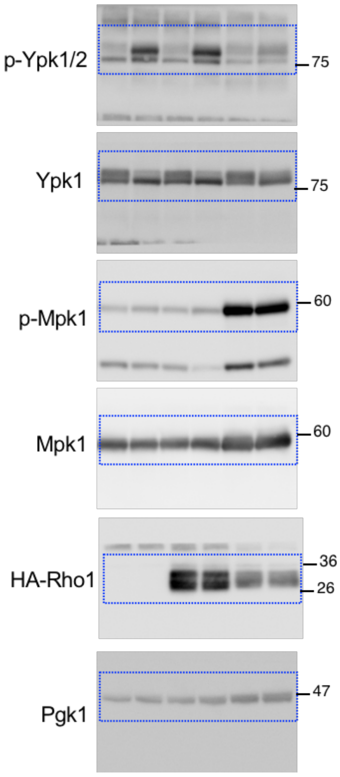

p-Ypk1/2
75
Ypk1
75
p-Mpk1
60
Mpk1
60
HA-Rho1
36
26
Pgk1
47


**Fig. S4**

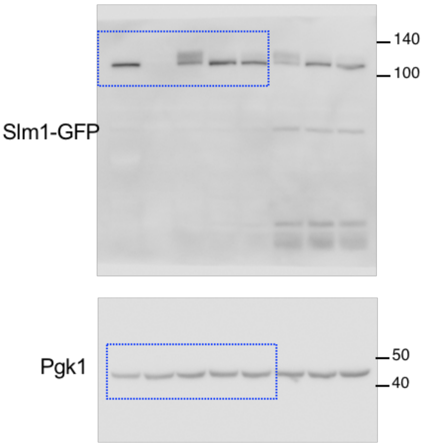

140
100
Slm1-GFP
Pgk1
50
40
