# Supplementary material for: Activation of the cell wall integrity pathway negatively regulates TORC2-Ypk1/2 signaling through blocking eisosome disassembly in Saccharomyces cerevisiae
Source: Commun Biol. 2024 Jun 11;7:722. doi: 10.1038/s42003-024-06411-2 (PMC11166964; doi:10.1038/s42003-024-06411-2)
Supplement: Supplementary file 3 — Description of additional supplementary files [file 42003_2024_6411_MOESM3_ESM.pdf]

Description of Additional Supplementary Files

File name: Supplementary Data 1

Description: Source data behind the graphs in the paper
